# Supplementary material for: A Glycemia-Based Nomogram for Predicting Outcome in Stroke Patients after Endovascular Treatment
Source: Brain Sci. 2022 Nov 18;12(11):1576. doi: 10.3390/brainsci12111576 (PMC9688182; doi:10.3390/brainsci12111576)
Supplement: Supplementary file 1 [file brainsci-12-01576-s001.zip › Table S2-three months mortality.pdf]

Table S2 Unadjusted and adjusted ORs of glycemia and other baseline characteristics for three-month mortality

	Crude OR (95% CI)	P	Adjusted OR (95% CI)	P
FBG	1.010(1.007-1.014)	<0.001	1.012(1.006-1.018)	<0.001
A/C glycemic ratio	9.473(4.941-18.164)	<0.001	4.783(2.183-10.478)	<0.001
Δ_{A-C}	1.015(1.010-1.019)	<0.001	1.007(1.002-1.011)	0.006
Age	1.041(1.024-1.059)	<0.001	1.036(1.014-1.057)	0.001
Atrial fibrillation	1.805(1.262-2.581)	0.001	0.775(0.475-1.264)	0.308
Serum creatinine	1.007(1.002-1.013)	0.006	1.006(1.001-1.011)	0.023
Baseline NIHSS score	1.108(1.082-1.136)	<0.001	1.093(1.063-1.123)	<0.001
Stroke subtypes	1.162(0.980-1.377)	0.083		
Infarct circulation	0.502(0.322-0.782)	0.002	0.619(0.343-1.117)	0.111
ASITN/SIR	0.467(0.357-0.611)	<0.001	0.638(0.468-0.870)	0.005
Door to first recanalization	1.001(1.000-1.003)	0.157		
Number of devices passed	1.349(1.183-1.539)	<0.001	1.279(1.087-1.504)	0.003
mTICI score	0.522(0.325-0.839)	0.007	0.911(0.719-1.155)	0.442

Adjusted for age, atrial fibrillation, serum creatinine, baseline NIHSS score, stroke subtypes, infarct circulation, ASITN/SIR, door to first recanalization time, number of devices passed and mTICI score

Abbreviations: OR, odds ratio; FBG, fasting blood glucose; A/C, FBG/chronic; Δ_{A-C}, the difference between FBG and chronic glycemia
