# Supplementary material for: A Glycemia-Based Nomogram for Predicting Outcome in Stroke Patients after Endovascular Treatment
Source: Brain Sci. 2022 Nov 18;12(11):1576. doi: 10.3390/brainsci12111576 (PMC9688182; doi:10.3390/brainsci12111576)
Supplement: Supplementary file 1 [file brainsci-12-01576-s001.zip › Table S3-sICH.pdf]

Table S3 Unadjusted and adjusted ORs of glycemia and other baseline characteristics for symptomatic intracranial hemorrhage

	Crude OR (95% CI)	P	Adjusted OR (95% CI)	P
FBG	1.010(1.005-1.015)	<0.001	1.009(1.003-1.015)	0.004
A/C glycemic ratio	9.293(3.856-22.398)	<0.001	5.096(1.804-14.392)	0.002
Δ_{A-C}	1.017(1.010-1.024)	<0.001	1.012(1.004-1.020)	0.003
Age	1.032(1.005-1.060)	0.018	1.005(0.975-1.036)	0.765
Atrial fibrillation	2.357(1.302-4.268)	0.005	0.998(0.477-2.090)	0.997
HDL	1.029(0.994-1.066)	0.106		
Baseline NIHSS score	1.046(1.012-1.082)	0.008	1.043(0.996-1.092)	0.076
Stroke subtypes	1.116(0.854-1.459)	0.420		
Infarct circulation	3.045(0.932-9.950)	0.065		
ASITN/SIR	0.439(0.293-0.658)	<0.001	0.517(0.334-0.800)	0.003
Onset to door time	0.997(0.995-0.999)	0.009	0.998(0.996-1.000)	0.078
Number of devices passed	1.382 (1.139-1.677)	0.001	1.257(1.018-1.552)	0.034

Adjusted for age, atrial fibrillation, HDL, baseline NIHSS score, stroke subtypes, infarct circulation, ASITN/SIR, onset to door time and number of devices passed

Abbreviations: OR, odds ratio; FBG, fasting blood glucose; A/C, FBG/chronic; Δ_{A-C}, the difference between FBG and chronic glycemia
