# Supplementary figures and images for: A Glycemia-Based Nomogram for Predicting Outcome in Stroke Patients after Endovascular Treatment
Source: Brain Sci. 2022 Nov 18;12(11):1576. doi: 10.3390/brainsci12111576 (PMC9688182; doi:10.3390/brainsci12111576)

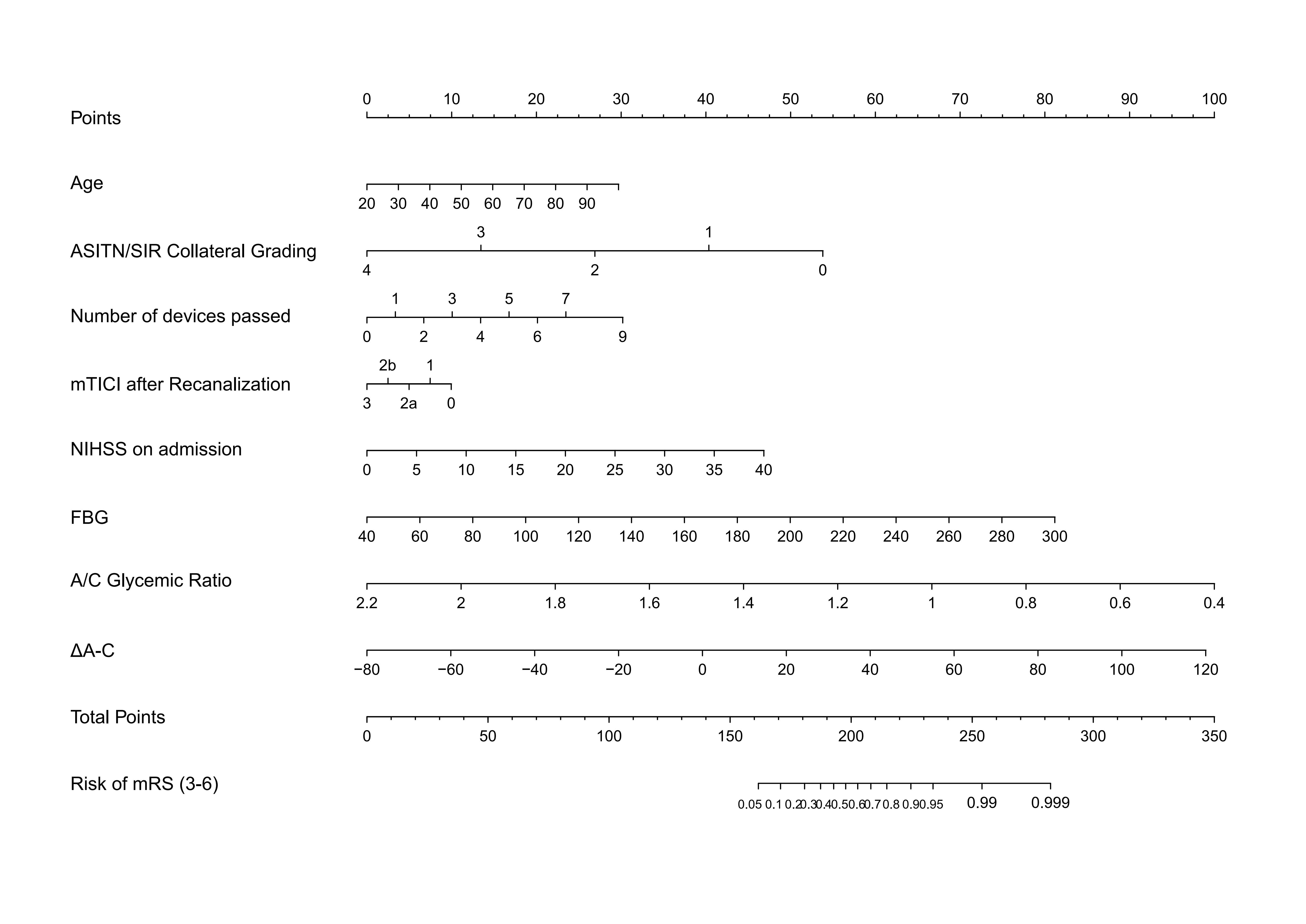

Supplement: Supplementary file 1 [file brainsci-12-01576-s001.zip › Figure S1.png]
